# Changes in segmentation and setation along the anterior/posterior axis of the homonomous trunk limbs of a remipede (Crustacea, Arthropoda)

**DOI:** 10.7717/peerj.2305

**Published:** 2016-08-10

**Authors:** Viacheslav N. Ivanenko, Ekaterina A. Antonenko, Mikhail S. Gelfand, Jill Yager, Frank D. Ferrari

**Affiliations:** 1 Department of Invertebrate Zoology, Biological Faculty, Lomonosov Moscow State University,Moscow,Russia; 2 Faculty of Mechanics and Mathematics, Lomonosov Moscow State University,Moscow,Russia; 3 A.A.Kharkevich Institute for Information Transmission Problems, Moscow, Russia; 4 Skolkovo Institute od Science and Technology, Skolkovo, Moscow Region, Russia; 5 Faculty of Bioengineering and Bioinformatics, Lomonosov Moscow State University, Moscow, Russia; 6 Department of Invertebrate Zoology, National Museum of Natural History, Smithsonian Institution, Washington D.C., USA; 7 McLean,VA,USA

**Keywords:** Remipedia, Crustacea, Arthropoda, Development, Thoracic limbs, Setation, Polynomial regressions, Vectors, Comparative morphology, Segmentation

## Abstract

This study describes the segmentation and setation at different developmental stages of the homonomous trunk limbs of the remipede *Speleonectes tulumensis* Yager, 1987 collected in anchialine caves of the Yucatan Peninsula. Most homonomous trunk limbs originate ventrolaterally and are composed of two protopodal segments, three exopodal segments and four endopodal segments; contralateral limb pairs are united by a sternal bar. However, the last few posterior limbs originate ventrally, are smaller sized, and have regressively fewer segments, suggesting that limb development passes through several intermediate steps beginning with a limb bud. A terminal stage of development is proposed for specimens on which the posterior somite bears a simple bilobate limb bud, and the adjacent somite bears a limb with a protopod comprised of a coxapod and basipod, and with three exopodal and four endopodal segments. On each trunk limb there are 20 serially homologous groups of setae, and the numbers of setae on different limbs usually varies. These groups of setae are arranged linearly and are identified based on the morphology of the setae and their position on the segments. The number of setae in these groups increases gradually from the anterior homonomous limb to a maximum between limbs 8–12; the number then decreases sharply on the more posterior limbs. Changes in the number of setae, which reach a maximum between trunk limbs 8–12, differ from changes in segmentation which vary only over the last few posterior trunk limbs. Following a vector analysis that identified a spatial pattern for these 20 groups of setae among the different homonomous limbs, the hypothesis was confirmed that the number of setae in any given group and any given limb is correlated with the group, with the position of the somite along the body axis, and with the number of somites present on the specimens. This is the first vector analysis used to analyze a pattern of developmental changes in serially homologs of an arthropod. Development of remipede limbs are compared and contrasted with similar copepod limbs. Architecture, particularly the sternal bar uniting contralateral limb pairs, proposed as homologous, and development of trunk limb segmentation of the remipede is generally similar to that of copepods, but the remipede limb differs in several ways including an additional endopodal segment, the proximal, that appears simultaneously with the protopod during development.

## Introduction

**Figure 1:**
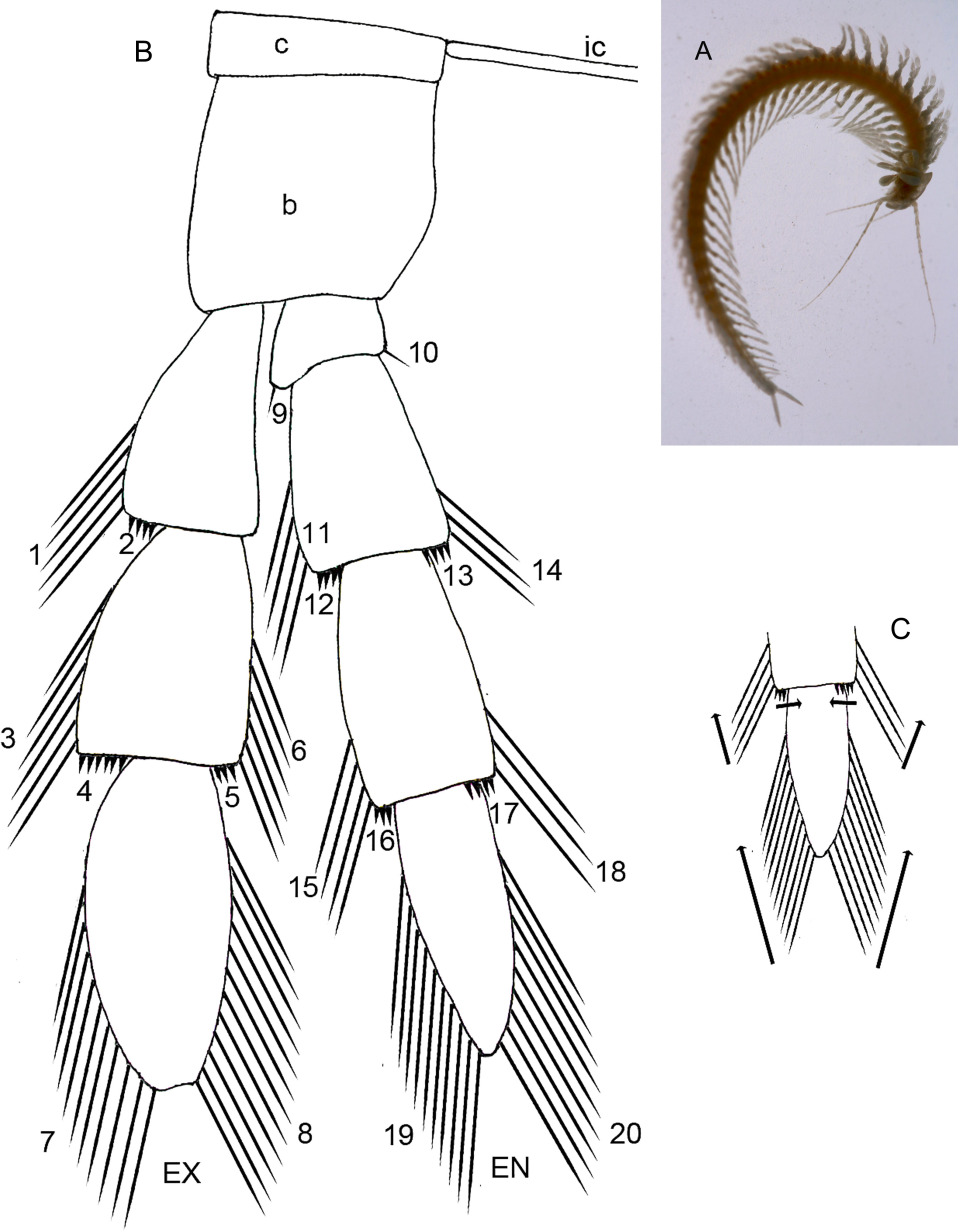
(A) Habitus of the remipede *Speleonectes tulumensis,* ventral view, scale bar 5 mm. Generalized schemes: (B) well-developed remipede homonomous limb (left) showing location of 20 groups of setae; (C) direction of addition of new setae along the rows. Abbreviations: c, coxapod; b, basipod; ic, intercoxal bar; ex, exopod; en, endopod. 1–20—groups of setae and spines.

Remipedia Yager, 1981 is a class of crustaceans discovered in anchialine caves and lava tubes within the last half century. To date, the relatively few extant species, about 28, have been placed in eight genera and three families. Morphological diversity is low among these crustaceans (Yager, 2013). Remipedes are characterized by a long body whose length results from a large number of trunk somites, at least sixteen in adults, a situation similar to anostracan and notostracan branchiopod crustaceans as well as other extant arthropods like myriapods and some extinct arthropods including trilobites. Trunk somites are added to the remipede body anteriorly from the anal somite; thus, developmental age of somites increases posterior to anterior. Remipedes are also distinctive because the trunk limbs posterior to a single maxilliped are homonomous, i.e., having similar structure and arranged serially along the anterior/posterior axis. They are flattened anterio-posteriorly to form a paddle-like appendage similar to the swimming legs of copepods. In addition, these homonomous limbs possess an unusually large number of setae in one or more well-defined setal groups arranged linearly on each ramal segment of the limb. A terminal stage of development has not been reported for remipedes. Juvenile remipedes are not well-known; a single description has been published (Koenemann et al., 2009), but because the description is not detailed it cannot add to the descriptions and analysis presented here. This paper analyzes differences in segmentation and setation of homonomous trunk limbs on a set of developmental stages of *Speleonectes tulumensis* Yager, 1987 (Fig. 1A); a remipede with a large number of somites.

## Material and Methods

Six specimens identified as *Speleonectes tulumensis* from the type locality, an anchialine cave near Tulum Pueblo, Quintana Roo, Mexico (USNM 233533, USNM 233534, USNM 1142736, USNM 1142747) were examined. All specimens were collected beneath the density interface in euhaline water of 34–35 ppm; the temperature beneath the density interface was about 25 ^o^C. Table 1 gives information about collection locality, collector, length and number of somites for each specimen examined. Specimens were identified as *S*.* tulumensis* based on the structure of cephalic appendages, maxilliped, and caudal rami, and assuming any slight changes were due to development.

**Table 1:** Specimens examined and the locality.

**Specimen**	**Body length (mm)**	**Number of thoracopods**	**Figure**	**Table**	**Locality**	**Data of sampling**	**Collector**
A	25	39	2, 3	2	TD	4 Mar. 1987	M. Madden
B	24	37	5A–C	S2	CA	Jul. 1988	L. Abele
C	22	36	5D–E	S2	CA	14 Jul. 1988	M. Madden
D	19	35	5F–G	S2	CA	14 Jul.1988	M. Madden
E	16	33	–	S2	CA	14 Jul. 1988	M. Madden
F	06	23	4	3	SC	22 Aug. 2000	M. Madden

**Notes.**

Abbreviations TD Temple of Doom, Sistema Sac Aktun CA Cenote Aktun Ha SC Sistema Chac Mool

Specimens were sorted by the number of trunk limbs, cleared and dissected in lactic acid, stained by adding a solution of chlorazol black E dissolved in 70% ethanol/30% de-ionized freshwater, and examined in glycerin with bright-field or differential interference optics. Drawings were made with a camera lucida. Morphological terms generally follow Ferrari & Dahms (2007); location of structures along the three limb axes follow Cohen (1993). A recent sequence analysis of the DNA molecule of remipedes (Hoenemann et al., 2013) resulted in the placement of *Speleonectes tulumensis* in the genus *Xibalbanus*. Because no apomorphies were proposed for *Speleonectes* or *Xibalbanus*, the name *Speleonectes tulumensis* is retained here.

In this study trunk somites (TS) are numbered beginning with the somite bearing the maxilliped (TS 1) and ending with the somite anterior to the telson. All trunk somites bear a trunk limb (TL), a set of articulating elements, or segments, aligned along the proximal/distal axis (Fig. 1A). Observations and analyses here are restricted to limbs posterior to TL 1, the maxilliped. With the exception of the reduced trunk limbs on the last few somites, the trunk limbs appear homonomous. They are arranged with a dorsal branch, the exopod and a ventral branch, the endopod, articulating on the basipod; proximal to the basipod is the coxapod which articulates with the somite. Trunk limbs are never composed of more than a 2-segmented protopod, a 3-segmented exopod and a 4-segmented endopod (Fig. 1B), here abbreviated 2 - 3 - 4. Trunk limb segments often bear hollow articulating extensions, the setal elements. The words “seta” and “spine” are used here arbitrarily to denote relatively long, flexible vs short, rigid elements. Each seta or spine on the exopod and endopod was assigned to one of 20 homologous groups based on location, size and shape (Figs. 1B and 3B–3F). Each group of setae or spines is either a proximo-distal or transverse linear arrangement (Figs. 1B and 1C). The setae were counted in each group of all limbs on the left side of the longest and the shortest specimens (A and F). The setae of the small, posterior limbs were counted from for both sides.

**Table 2:** *Speleonectes tulumensis* Yager, 1987. Specimen A. Number of setal elements in each of 20 groups of the thoracopods 2–39 (TL).

	Exopod								Endopod												
	Proximal segment		Middle segment				Distal segment		Proximal segment		Mid Proximal segment				Mid Distal segment				Distal segment		
TL ∖ setal group	1	2	3	4	5	6	7	8	9	10	11	12	13	14	15	16	17	18	19	20
2r	8	2	8	3	2	7	13	10	2	2	2	3	2	6	4	3	2	7	7	10
3r	7	4	9	6	3	7	15	13	1	2	2	3	3	6	5	4	3	8	9	13
4r	6	5	9	9	4	7	16	13	0	1	2	5	2	5	5	5	3	8	11	14
5r	6	6	10	8	4	7	17	14	0	1	2	5	3	5	6	6	4	10	10	14
6r	7	6	10	10	2	7	18	15	0	1	1	3	3	4	5	6	4	8	11	14
7r	7	5	10	12	2	8	18	15	0	1	2	6	3	5	6	7	4	9	12	15
8r	6	5	10	10	0	7	19	16	0	1	1	6	2	4	5	6	4	8	12	15
9r	6	5	10	11	0	8	19	16	0	1	0	7	2	5	6	6	5	9	12	15
10r	5	6	10	11	0	8	20	16	0	1	1	6	2	5	6	6	5	8	12	15
11r	6	5	9	11	1	8	19	15	0	0	2	5	2	5	6	7	5	8	12	15
12r	6	5	10	10	0	8	20	16	0	1	1	6	2	3	5	6	4	7	12	15
13r	6	5	10	11	0	8	18	15	0	1	1	6	2	4	5	7	5	7	12	16
14r	6	5	10	11	0	7	20	15	0	1	0	6	1	3	6	6	4	8	12	15
15r	6	6	10	11	0	8	18	15	0	1	0	5	2	3	4	7	5	7	10	13
16r	5	5	10	11	0	7	18	15	0	0	0	5	2	3	3	7	4	6	13	14
17r	5	5	10	11	0	7	18	14	0	0	0	5	2	4	3	7	4	6	11	14
18r	5	5	9	11	0	8	17	14	0	0	0	6	1	2	4	5	5	7	11	13
19r	5	5	9	11	0	7	17	15	0	0	0	5	2	2	3	7	4	6	10	14
20r	4	5	9	12	0	7	17	14	0	0	0	6	2	2	3	7	4	5	10	13
21r	4	5	9	10	0	7	16	14	0	0	0	6	1	2	3	6	4	5	9	12
22r	4	5	9	10	0	7	16	14	0	0	0	5	1	2	3	6	4	6	10	12
23r	4	5	9	10	0	7	16	14	0	0	0	5	1	2	3	6	4	6	10	12
24r	4	5	9	10	0	7	16	14	0	0	0	6	1	3	3	7	4	5	10	12
25r	3	4	8	10	0	7	17	14	0	0	0	5	1	2	3	6	4	4	9	13
26r	4	4	8	9	0	7	16	14	0	0	0	5	1	2	2	6	4	4	9	11
27r	3	4	8	11	0	5	16	13	0	0	0	5	1	2	2	6	4	4	9	11
28r	3	4	7	9	0	6	15	13	0	0	0	5	0	1	2	6	4	4	8	11
29r	3	4	7	9	0	6	13	13	0	0	0	5	0	1	2	6	4	3	7	10
30r	2	3	6	8	0	6	13	12	0	0	0	5	0	1	1	6	3	3	7	10
31r	2	2	6	10	0	5	12	12	0	0	0	4	0	1	1	6	3	2	8	9
32r	2	2	6	8	0	5	13	11	0	0	0	3	0	0	1	5	3	2	8	9
33r	2	2	5	8	0	3	11	11	0	0	0	3	0	0	1	5	3	2	6	9
34r	1	0	4	7	0	5	10	11	0	0	0	1	0	0	1	4	0	0	7	7
35r	1	0	3	5	0	4	9	10	(-)	(-)	0	1	0	0	1	3	1?	0	7	7
36r	1	0	2	3	0	2	8	9	0	0	0	1	0	0	1	1	0	0	5	4
36l	1	0	1	0	0	1	5	6	0	0	0	1	0	0	1	0	0	0	2	3
37r	0	0	2	2	0	1	7	6	0	0	0	1	0	0	1	0	0	0	3	3
38r	0	0	1	0	0	1	3	4	0	0	0	1	0	0	0	0	0	0	1	2
39l (bud)	(-)	(-)	(-)	(-)	(-)	(-)	(-)	1?	(-)	(-)	(-)	(-)	(-)	(-)	(-)	(-)	(-)	(-)	(-)	0?

**Notes.**

Abbreviations (-) the segment is absent r right leg l left leg

**Table 3:** *Speleonectes tulumensis* Yager, 1987. Specimen F. Number of setal elements in each of 20 groups of the thoracopods 2–23 (TL).

TL ∖ setal group	1	2	3	4	5	6	7	8	9	10	11	12	13	14	15	16	17	18	19	20
2r	2	1	2	1	0	1	3	3	0	1	0	1	0	1	1	0	0	1	1	3
2l	2	1	2	1	0	1	4	3	0	1	0	1	0	1	1	0	0	1	2	3
3r	2	1	2	1	0	1	4	3	0	1	0	1	1	1	1	0	1	1	2	4
4r	2	1	2	2	0	1	4	4	0	1	0	1	0	1	1	1	1	2	2	4
5r	2	1	2	2	0	1	5	4	0	1	0	1	1	1	1	0	1	2	2	4
6r	2	0	2	2	0	2	5	4	0	1	0	1	1	1	1	1	1	2	3	4
7r	2	0	2	2	0	2	5	5	0	1	0	1	0	1	1	0	1	2	3	4
8r	2	0	2	2	0	2	5	5	0	1	0	1	0	1	1	0	1	2	3	4
9r	1	1	2	2	0	2	5	4	0	1	0	0	0	1	1	0	1	2	3	4
10r	1	0	2	2	0	2	4	4	0	1	0	1	0	1	1	0	1	2	3	4
11r	1	0	2	1	0	2	4	4	0	1	0	1	0	1	1	0	1	2	2	4
12r	1	0	2	2	0	1	4	4	0	1	0	1	0	1	1	0	1	2	2	4
13r	1	0	2	1	0	1	4	3	0	0	0	1	0	1	1	0	0	2	2	4
14r	1	0	2	1	0	1	4	3	0	0	0	1	0	1	1	0	0	1	2	3
15r	1	0	2	1	0	1	3	3	0	0	0	1	0	1	1	0	0	1	2	3
16r	1	0	2	1	0	1	3	3	0	0	0	0	0	1	0	0	0	1	1	3
17r	1	0	1	1	0	1	3	2	0	0	0	0	0	1	0	0	0	1	2	3
18l	1	0	1	0	0	1	3	2	0	0	0	0	0	1	0	0	0	1	1	2
19r	1	0	1	0	0	1	2	2	0	0	0	0	0	1	0	0	0	1	1	2
20r	1	0	1	0	0	0	2	2	0	0	0	0	0	0	(-)	(-)	(-)	(-)	1	2
21r	(-)	(-)	(-)	(-)	(-)	(-)	1	1	0	0	(-)	(-)	(-)	(-)	(-)	(-)	(-)	(-)	1	1
22l	(-)	(-)	(-)	(-)	(-)	(-)	0	1?	(-)	(-)	(-)	(-)	(-)	(-)	(-)	(-)	(-)	(-)	0	0
23l (bud)	(-)	(-)	(-)	(-)	(-)	(-)	(-)	1?	(-)	(-)	(-)	(-)	(-)	(-)	(-)	(-)	(-)	(-)	(-)	0?

**Notes.**

Abbreviations (-) the segment is absent r right leg l left leg

Counts of seta per group on each trunk limb were placed in a matrix (Tables 2 and 3). The matrix *A* = (*a*_*i*,*j*_) represents the setation of one specimen, where *a*_*i*,*j*_ is the number of setae in the group* j* of the trunk limb *i*. We considered a hypothesis that the data in the matrix may be compressed, and the setation of a given group in a given limb depends only on the group and limb identity, without any additional interaction. Decomposition of the matrix *A* into the product of vectors *x* = (*x*_*i*_) and *y* = (*y*_*j*_) reflecting setation of a trunk limb and setation of a particular group of setae, respectively, was performed by minimization of the residual ∥*A*–*Â*∥, where *Â* = *x*^*T*^⋅ *y* is an approximation matrix of rank one and *x*^*T*^ is a column vector that means transposed vector *x*. The matrix norm here is given by ∥*A*∥ = Σ_*i*,*j*_(*a*_i,j_)^2^. Calculations were done using a one-rank approximation of matrix *A* (see Supplemental Information 1). The vectors *x* and *y* are defined accurate within a multiplication constant, so they were normalized to length 1 with the same norm ∥*x*∥ = Σ_*i*_(*x*_i_)^2^. This normalization introduces an additional multiplicative coefficient *c*, thus *Â* = *c* ⋅ *x*^*T*^ ⋅ *y*; ∥*x*∥ = ∥*y*∥ = 1. The coefficient *c* is expected to reflect the age of a specimen, whose proxy here is the number of somites. A polynomial regression was calculated using fitted linear models in R. The polynomial degree was selected based on the ANOVA method in R.

**Figure 2:**
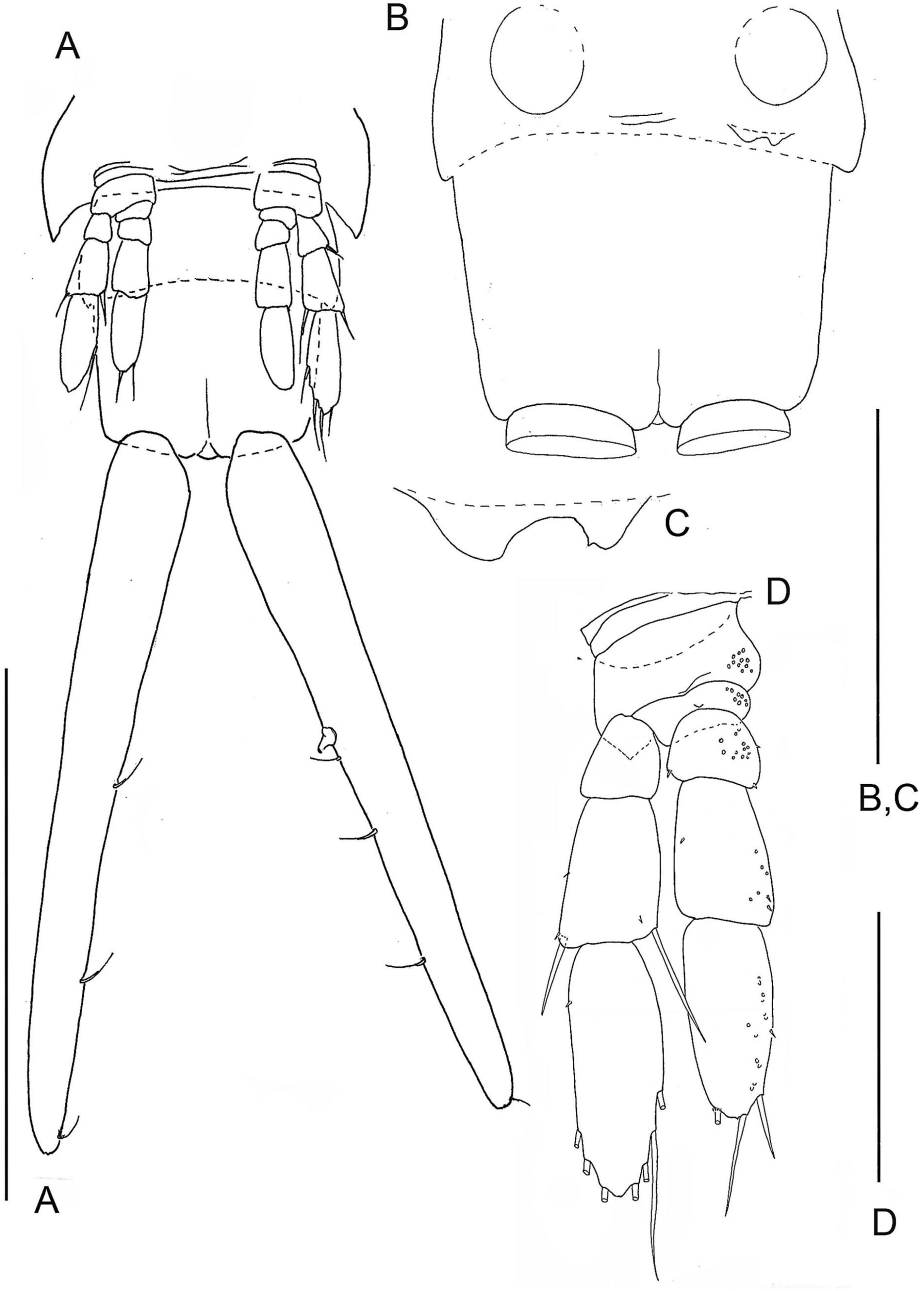
The remipede *Speleonectes tulumensis.* Specimen A. (A) Posterior trunk limbs, showing ventral origin, anal somite and caudal ramus; (B) posterior TS 39 with left limb bud and anal somite; (C) left limb bud, right bud is absent; (D) Left TL 38 as on (A). Scale bars: (A) 2 mm; (B, D) 0.4 mm; (C) 0.2 mm.

**Figure 3:**
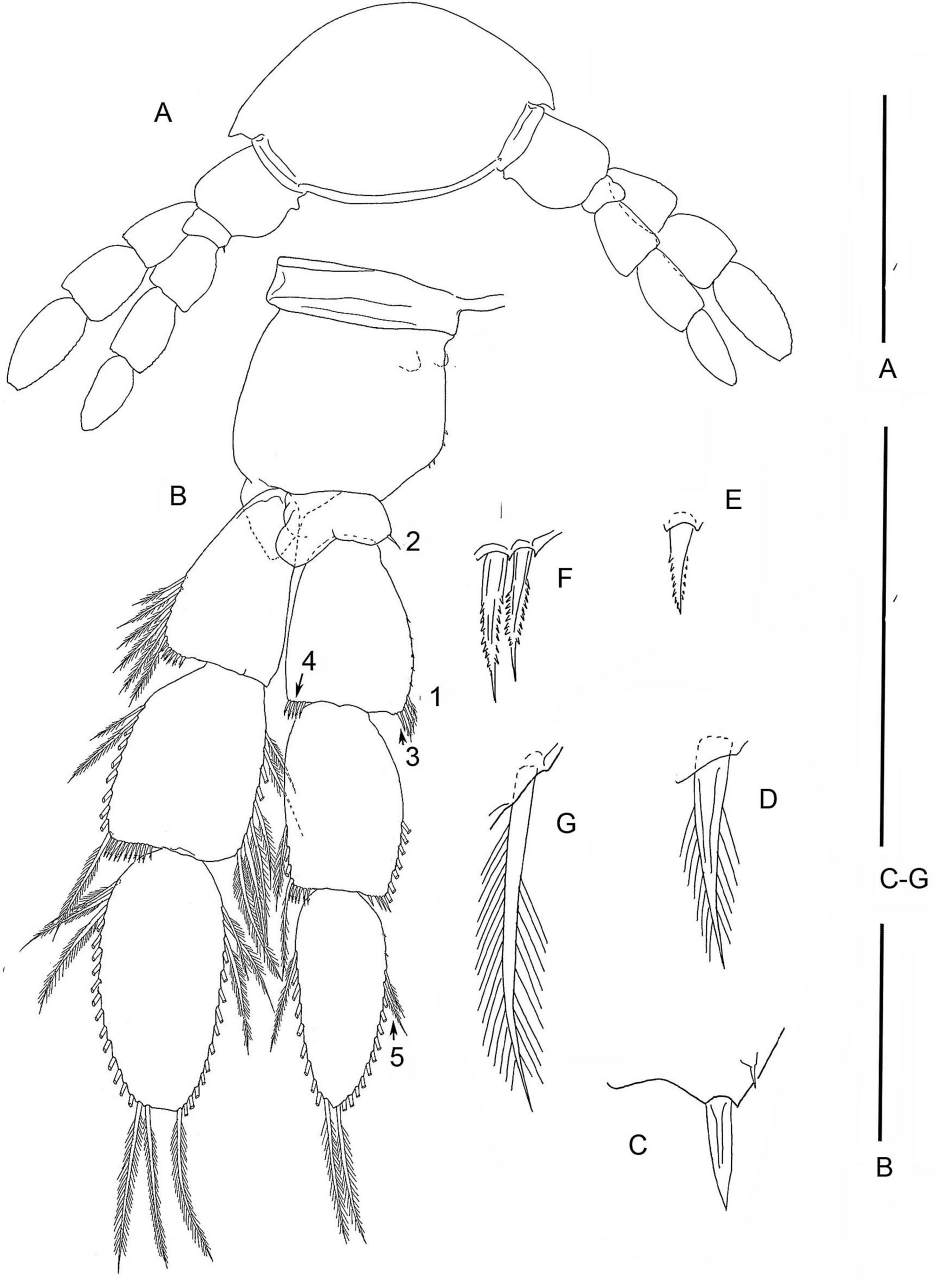
The remipede *Speleonectes tulumensis.* Specimen A. (A) TL 15, showing a ventrolateral origin of more anterior limb; (B) Right TL 15, 1–4 indicating location of setal elements of different shapes (C–G) respectively. Scale bars: (A) 2 mm; (B) 0.8 mm; (C–G) 0.4 mm.

**Figure 4:**
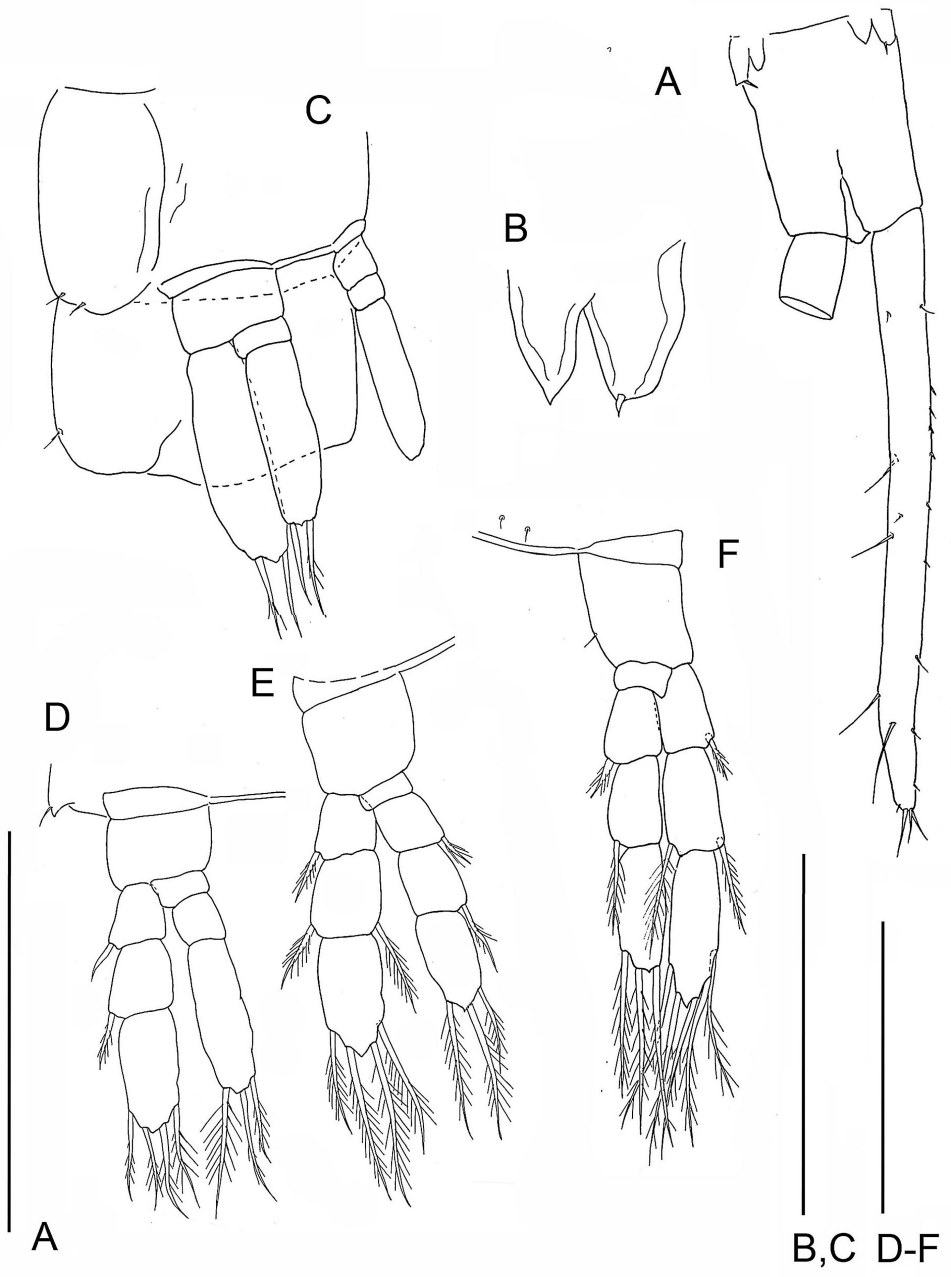
The remipede *Speleonectes tulumensis.* Specimen F. (A) limb bud on TS 23 and anal somite with caudal ramus; (B) limb bud of TS 23; (C) TS 22 and 23 with TL 22; (D) TL 21; (E) TL 20; (F) TL 19. Scale bars: (A) 0.8 mm; (B) 0.2 mm; (C–F) 0.4 mm.

**Figure 5:**
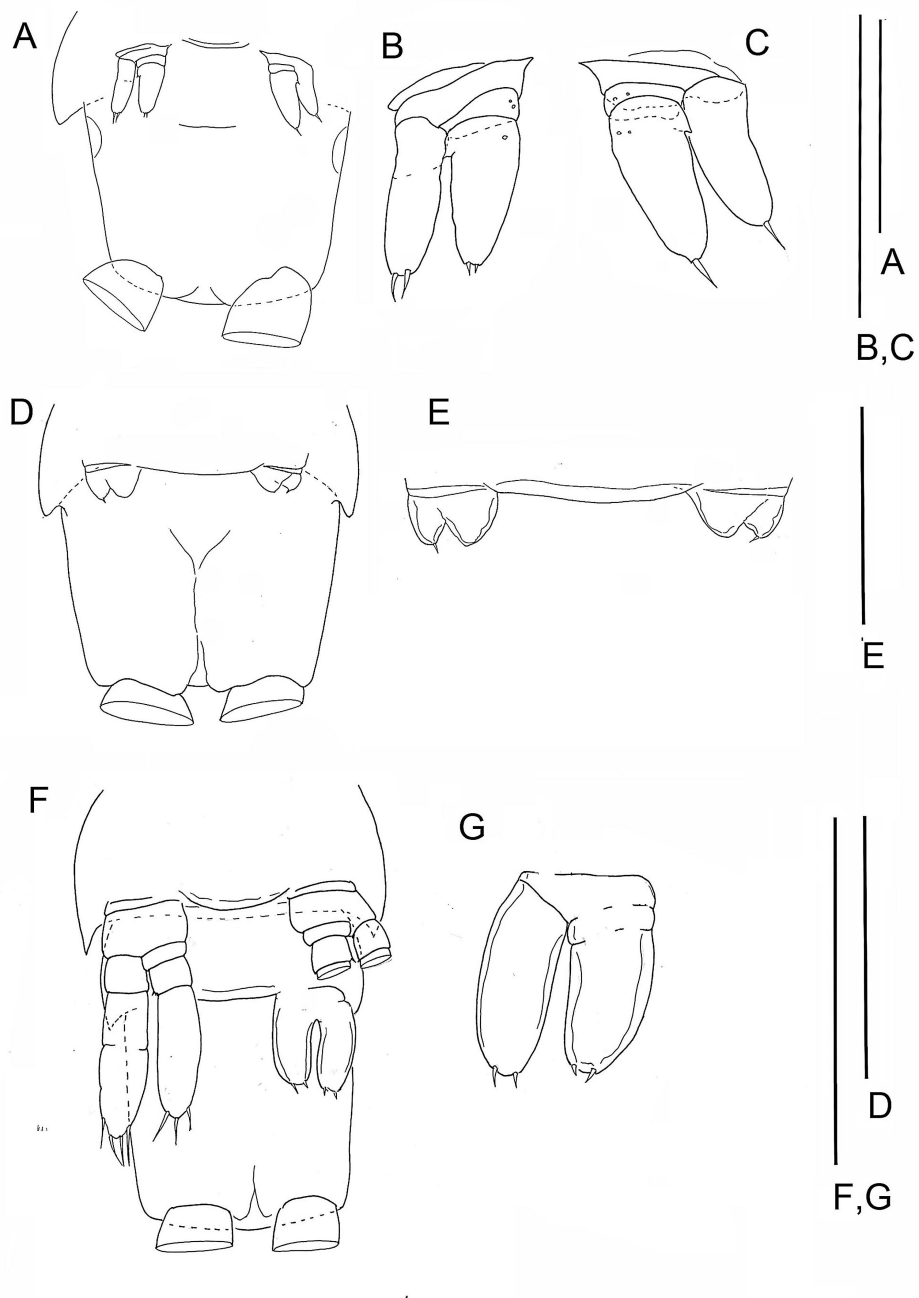
The remipede *Speleonectes tulumensis.* Specimen B: (A) TS 37 plus anal somite; (B) right TL 37; (C) left TL37. Specimen C: (D) TS36 plus anal somite; (E) limb buds of TL 36. Specimen E: (F) TS 32 with TL32, TS33 with left bud of TL 33 plus anal somite; (G) limb bud of right TL 33. Scale bars: (A, D, F) 0.8 mm; (B, C, G) 0.4 mm; (E) 0.2 mm.

**Figure 6:**
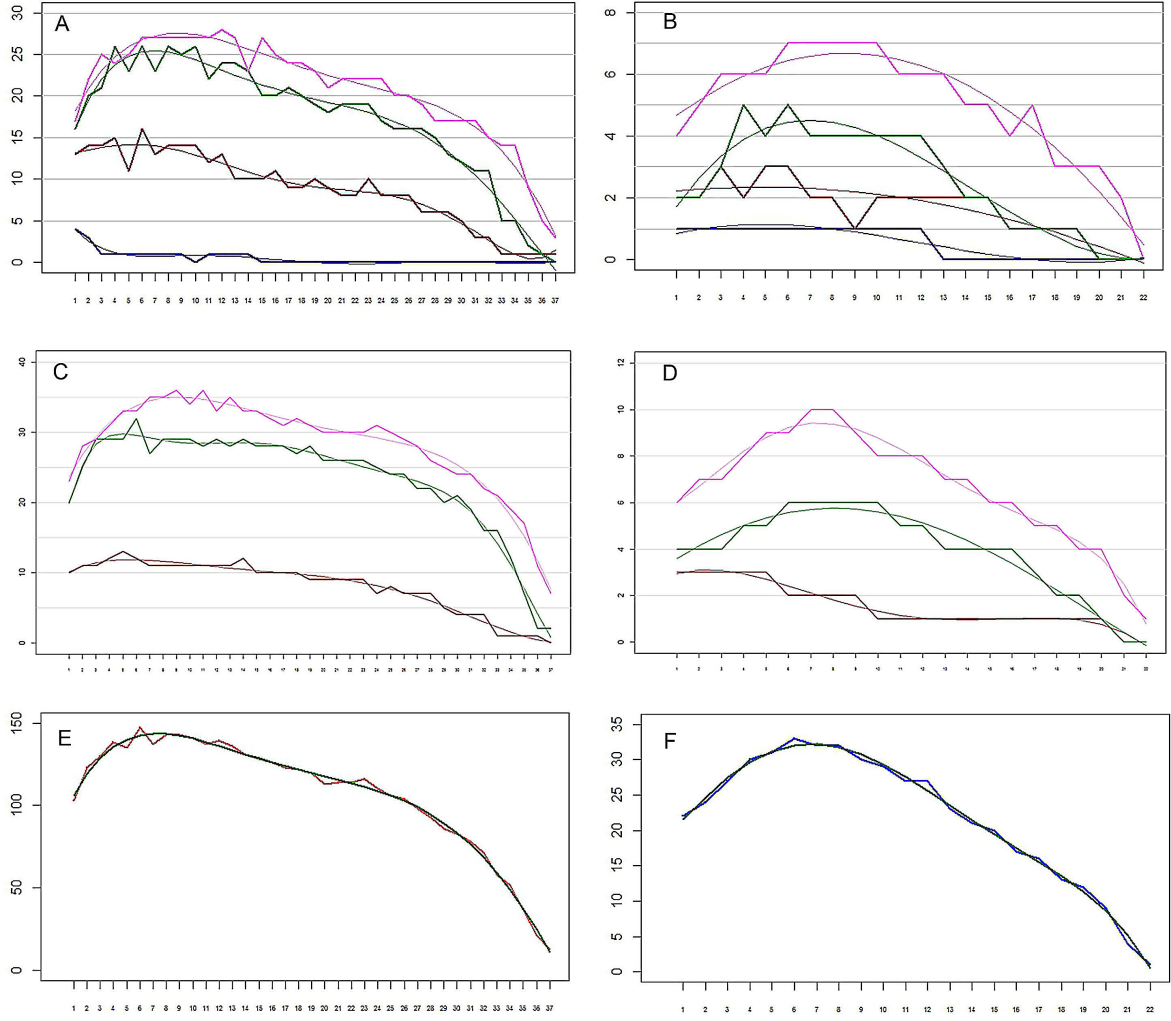
The remipede *Speleonectes tulumensis.* (A) Specimen A, Endopod. Setation of four endopodal segments and corresponding regression trend lines. (B) Specimen F, Endopod. Setation of four endopodal segments and corresponding regression trend lines. (C) Specimen A, Exopod. Setation of three exopodal segments and corresponding regression trend lines. (D) Specimen F, Exopod. Setation of three exopodal segments and corresponding regression trend lines. (E) Specimen A. Setation of segments and the polynomial regression trend line with the best fitted coefficients. (F) Specimen F. Setation of segments and the polynomial regression trend line with the best fitted coefficients. Endopod: pink—distal segment (groups 19–20), green—mid distal segment (groups 15–18), brown—mid proximal segment (groups 11–14), blue—proximal segment (groups 9–10). Exopod: Pink—distal segment (groups 7–8), green—middle segment (groups 3–6), brown—proximal segment (groups 1–2). Abbreviations: young, specimen F; old, specimen A; real, observed; sum, total of all segments.

## Results

### Morphology

(Figure 2–6, Tables 2–4; Table S1–S5)

Most homonomous trunk limbs, i.e., those posterior to the maxilliped, on *S. tulumensis* are segmented 2 - 3 - 4 (Figs. 2A and 3A), and most of these limbs originate ventrolaterally on their somite (Fig. 3A). The coxapod of the protopod is thin, asetose and united to its contralateral twin by a thin transverse ligamentous bar (Hessler & Yager, 1998), also referred to as a sternal bar (Koenemann, Schram & Iliffe, 2006). The morphology of this bar does not vary among limbs of *S*. *tulumensis* as it may among other remipedes (Koenemann, Schram & Iliffe, 2006), although it often varies in length. The last few posterior limbs originate ventrally (Fig. 2A) and have a sternal bar that is short (Fig. 4C) relative to the anterior limbs (Fig. 3A). The basipod of the limb is larger than the coxapod, about as long as deep; proximoventrally there may be knobs anteriorly and posteriorly and a few denticles ventrally, but there are no setae. The proximal endopodal segment is small and has a distinctive distoventral lobe. The two middle segments of the endopod and the middle segment of the exopod are subquadrate; the proximal segment of the exopod broadens distally (Fig. 3A). The distal segment of both rami is ovate. Setae occur only on the rami, and can be assigned to groups as shown in Fig. 1B*.*

The last few posterior trunk somites bear limbs that are reduced in size and segmentation; these limbs originate ventrally on their somite rather than ventrolaterally (Fig. 2A). The morphology of these posterior limbs varies among different specimens. For example, on the specimen with 39 trunk somites plus telson (specimen A), the posterior trunk limb, TL 39, appears as an unarticulated, unarmed, sclerotized bilobate bud present only on the left side (Fig. 2B). The lobes are the presumptive rami but the limb lacks a distinct protopod. The somite having this bud has a pair of circular sclerotizations with unknown function and not found on other examined specimens. TL 38, immediately anterior on this specimen, has 2 - 3 - 4 segments (Fig. 2D).

On the specimen B with 37 trunk somites plus telson TL 37 is not a bud; its segmentation is 2 - 1 - 2. Each ramus of one limb bears two apical setae; the contralateral pair bears only 1 apical seta. The proximal, unarmed segment of the 2-segmented endopod does not exhibit the distinctive distoventral lobe found on the proximal segment of a 4-segmented endopod of more developed limbs (Fig. 3B).

**Table 4:** Regression polynomials for setation vectors of limbs. *j* is a limb number. Polynomials are given for segments separately (Figs. 6A–6D), for sums of setae in segments (Figs. 6E and 6F), for segment vectors (Figs. 8A and 8B) and for vectors for limbs for specimens A and F given in one scale (Fig. 7C).

Ramus	Corr. Segment number	Segment	Specimen A	Specimen F
Exopod	1	Proximal	10.58 + 0.26⋅*j* – 0.022⋅*j*^2^+ 0.0004⋅*j*^3^	2.55 + 0.52⋅*j* – 0.14⋅*j*^2^ + 0.01⋅*j*^3^ – 0.0002⋅ x^4^
	2	Middle	20.06 + 2.90⋅*j* – 0.29⋅*j*^2^ + 0.011⋅*j*^3^ – 0.0002⋅*j*^4^	2.94 + 0.67⋅ x – 0.034⋅ x^2^ – 0.0011⋅ x^3^
	3	Distal	19.74 + 4.29⋅*j* – 0.41⋅*j*^2^ + 0.015⋅*j*^3^ – 0.0002⋅*j*^4^	3.79 + 1.98⋅ x – 0.24⋅ x^2^ + 0.011⋅ x^3^ – 0.0002⋅ x^4^
Endopod	0	Proximal	3.70 – 0.65⋅*j* + 0.047⋅*j*^2^ – 0.0015⋅*j*^3^	1.19 – 0.035⋅*j* – 0.0014⋅*j*^2^
	1	Mid Proximal	13.51 + 0.26⋅*j* – 0.049⋅*j*^2^ + 0.0017⋅*j*^3^	2.16 + 0.078⋅*j* – 0.0083⋅*j*^2^
	2	Mid Distal	14.70 + 3.11⋅*j* – 0.31⋅*j*^2^ + 0.011⋅*j*^3^ – 0.0001⋅*j*^4^	2.48 + 0.36⋅*j* – 0.024⋅*j*^2^
	3	Distal	14.91 + 3.66⋅*j* – 0.35⋅*j*^2^ + 0.013⋅*j*^3^ – 0.0002⋅*j*^4^	4.11 + 0.60⋅*j* – 0.035⋅*j*^2^
		Sum	89.48 + 19.00⋅*j* – 2.2757⋅*j*^2^ + 0.11⋅*j*^3^ – 0.0025⋅*j*^4^	18.27 + 3.21⋅*j* + 0.11⋅*j*^2^ - 0.066⋅*j*^3^ + 0.0043⋅*j*^4^ – 0.0001⋅*j*^5^
		Vectors for all limbs		
			0.16 – 0.21⋅*j* – 0.16⋅*j*^2^ – 0.004⋅*j*^3^ – 0.059⋅*j*^4^	0.2 – 0.27⋅*j* – 0.22⋅*j*^2^ + 0.04⋅*j*^3^ – 0.028⋅*j*^4^ – 0.029⋅*j*^5^
		Common	0.71 + 0.14⋅*j* – 0.012⋅*j*^2^	

**Figure 7:**
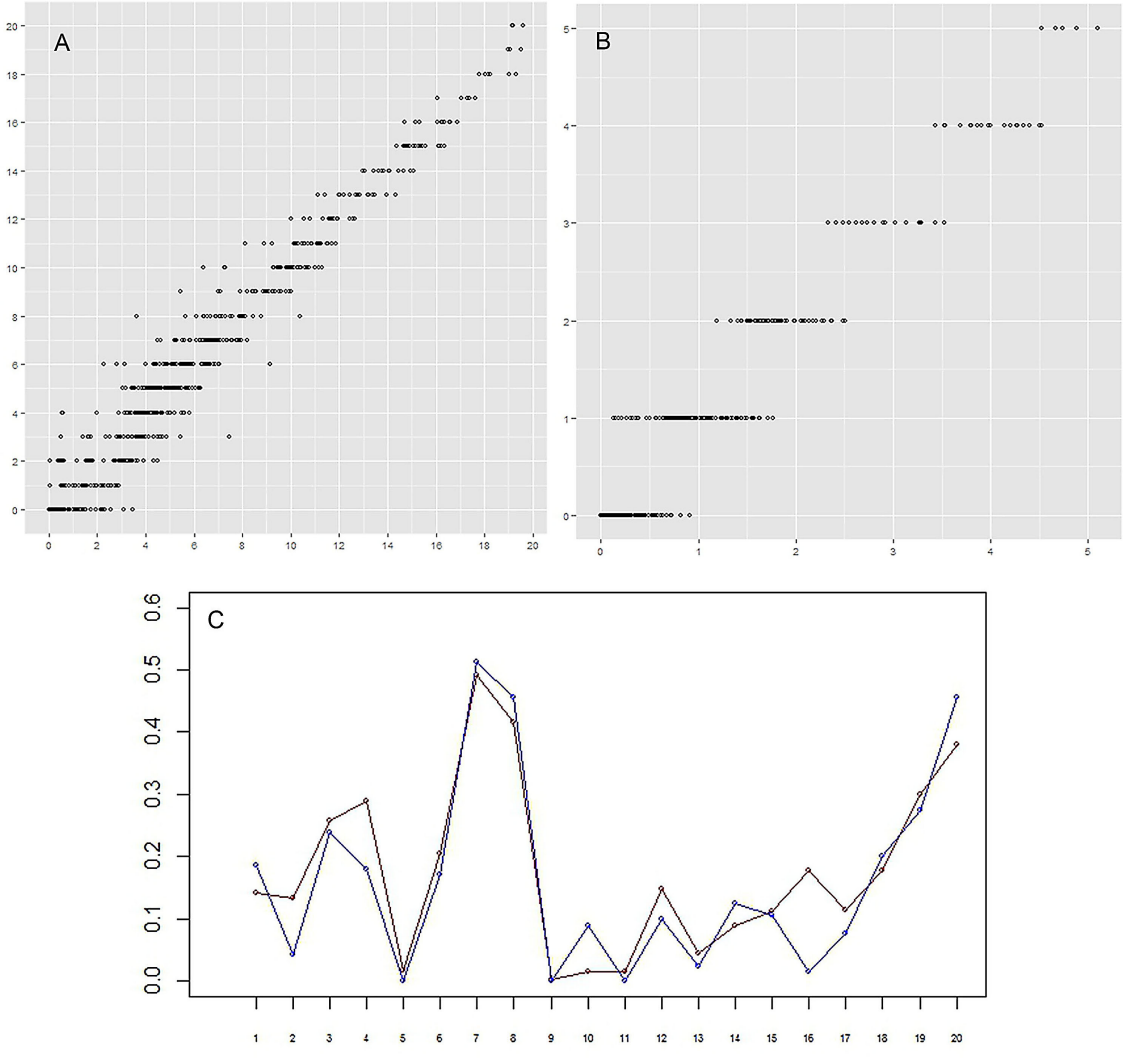
(A–B) Correlation between the actual number of setae and the predicted number. For every group of setae and every limb (that is one point on plot) the real (vertical axis) and approximated (horizontal axis) number of setae are given. (A) (left)–specimen A; (B) (right)–specimen F. The correlation between real data and prediction is 0.98 and 0.97 for specimens A and F, respectively, *p*-value < 2.2e–16. (C) Setation vectors *y* = (*y*_j_) for groups. Red: specimen A; blue: specimen F. The correlation between two vectors is equal to 0.93. Horizontal axis: numerical idenficator *j* of the group. Vertical axis: the value of the setation vector component, *y*_*j*_.

The posterior limb on specimen C with 36 trunk somites plus telson appears as a bilobate bud which articulates proximally (Figs. 5D and 5E); there is no evidence of a protopod. The segmentation of the adjacent TL 35 is 2 - 3 - 4. On specimen D with 35 trunk somites plus telson, the posterior limb, TL 35, has 2 - 3 - 4 segments. On specimen E with 33 trunk somites plus telson the posterior limb, TL 33, is 2 - 1 - 2 (Figs. 5F and 5G). The middle and distal exopodal segments on the adjacent TL 31 are incompletely articulated (Fig. 5F).

On the smallest examined specimen, specimen F, with 23 trunk somites plus telson, TL 23 is an unarticulated bilobate bud with 1 terminal seta on the presumptive exopod; the presumptive endopod is unarmed (Fig. 4B), and a protopod has not formed. This limb is similar to the posterior limb on the specimen with 36 trunk somites. The limb on the adjacent TS 22 has a 2 - 0 - 2 segments (Fig. 4C); the proximal endopodal segment is a simple, unarmed cylinder without a distinctive distoventral lobe. This limb is not as well-developed as the second to posterior limb on the specimen with 39 trunk somites or with 32 trunk somites.

On the homonomous limbs, each of 20 setal group**s** is represented by a linear arrangement of setae (groups 1, 3, 7–9, 11, 14, 15, 18–20) or spines (groups 2, 4, 5, 12, 13, 16, 17) except groups 9 and 10 that have no more than two seta (Figs. 1B, 3, Tables 2 and 3). The setae of most limbs, except the more posterior limbs, bear lateral setules, solid extensions of the epicuticle. The number of setae and spines may be distinct for different setal groups, and usually varies from 0 to 20 on limbs of different specimens (Tables 2 and 3). The total number of setae on the limbs, as well as the number on most of the setal groups (with the exceptions of groups 9 and 10 on the proximal endopodal segment) increases from the somite bearing TL 2 to the somite bearing TL 7. Posterior to TL 8 these setal numbers decrease progressively. This decrease in the number of setae on posterior limbs of the largest specimen, specimen A with 39 trunk limbs, is more pronounced than that of the specimen F, the smallest, with 21 trunk limbs (Tables 2 and 3). Setae or spines belonging to groups 2, 4, 5, 9, 10, 12–14, 16 and 17 are not present in the smallest specimen F (Fig. 4F). Other setal groups are absent on more posterior limbs of different specimens (Tables 2 and 3). Patterns of setation of all endopodal and exopodal segments, and corresponding regression trend lines are given in Fig. 6 and Table 4.

### Decomposition of the setation matrix into the trunk limb and group setation vectors

From the above observations, a hypothesis was considered that the number of setae in a given group and on a given limb is defined by three factors: the setal group on the segment, the location of the limb along the body axis, and the total number of somites, the later being a proxy for the age. No interaction terms were included. To test this hypothesis, the setation matrix was decomposed into the product of group and limb vectors (see Methods).

The correlation between the actual number of setae and the predicted number is very high, 0.98 and 0.97 for specimens A and F, respectively (Figs. 7A and 7B). These two specimens were selected for comparison because the body of specimen A was comprised of the largest number of somites and specimen F the smallest number. The setation vectors for groups are similar for the two specimens (Fig. 7C), the correlation coefficient being 0.93.

**Figure 8:**
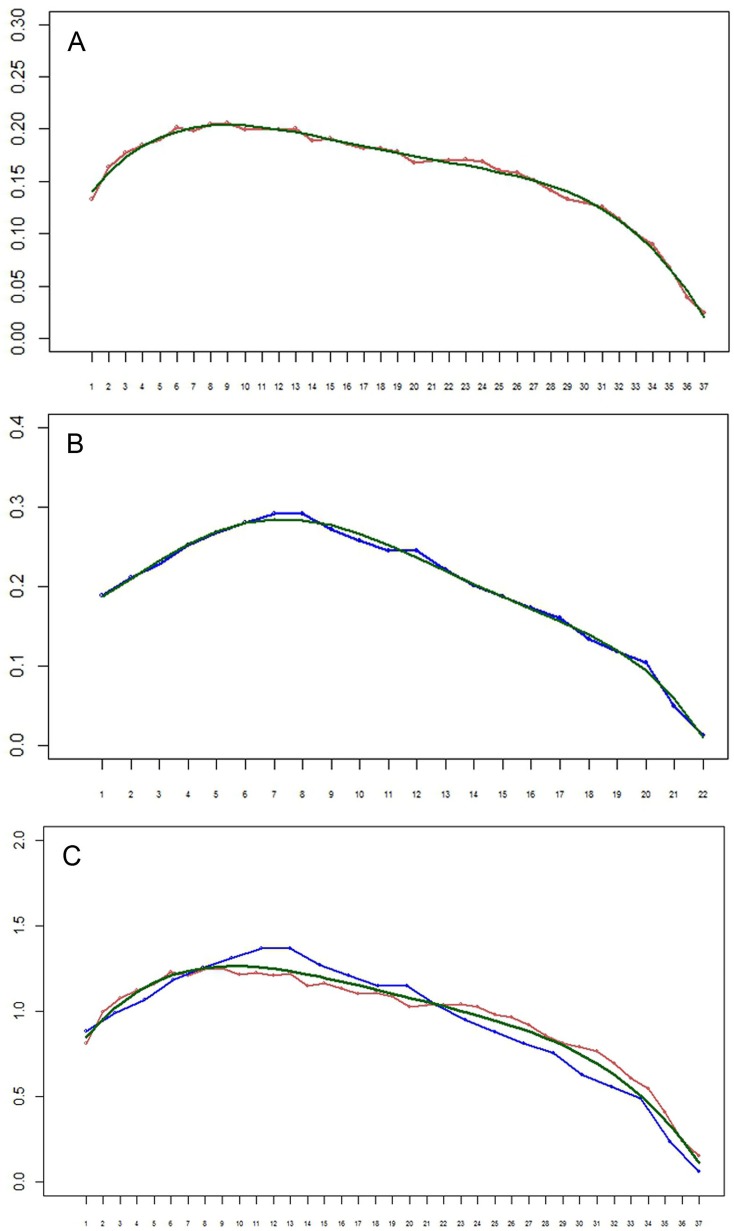
(A–B) Setation vectors *x*= (*x*_i_) for limbs of the remipede *Speleonectes tulumensis*. (A) specimen A, (B) specimen F. The polynomial regression trendlines (0.158 − 0.212*i* − 0.163*i*^2^ − 0.004*i*^3^ − 0.059*i*^4^ for specimen A, and 0.2 − 0.271*i* − 0.217*i*^2^ + 0.04*i*^3^ − 0.028*i*^4^ − 0.029*i*^5^ for specimen F) are given in green. Horizontal axis: limb number *i*. Vertical axis: the value of the setation vector component, *x*_i_. (C) Setation vectors for limbs in one scale. Horizontal axis: limb number *i*. Vertical axis: the value of the setation vector component, *x*_i_. Coordinates of limbs in specimen F are stretched in such a manner that length of vector, in effect the number of limbs, for specimen F matches that of specimen A. Red, specimen A; blue, specimen F; green, the polynomial regression trend line (0.713 + 0.143*i* − 0.012*i*^2^).

The setation vectors for limbs are shown in Fig. 8 (A, B) together with polynomial trend lines. Because the number of limbs of the two specimens is different, these vectors are not directly comparable. However, scaling the number of limbs to the same specimen length yields almost a perfect fit (Fig. 8C). The obtained curves demonstrate a loosely defined maximum around the first third of the interval, similar to the total number of setae.

## DISCUSSION

A terminal stage of development has not been proposed for remipedes. Evidence for such a stage of *S*. *tulumensis* was considered from morphology of the posterior trunk limb and the trunk limb on the adjacent somite. The terminal stage of a remipede is expected to have a simple bilobate limb bud on the last somite (e.g., Fig. 2B, specimen with 39 trunk limbs). The limb on the adjacent somite is expected to have a protopod with coxapod and basipod, and 3 exopodal and 4 endopodal segments (e.g., Fig. 2A).

In contrast, on a remipede hypothesized to undergo several more molts, the limb on the posterior somite is expected to express different limb morphologies among specimens. These include an elongate limb bud (e.g., Fig. 4, specimen with 23 trunk limbs; Figs. 5F–5G, specimen with 33 trunk limbs) or a transformed limb that has fewer than 3 exopodal and 4 endopodal segments, (e.g., Figs. 5A–5C, specimen with 37 trunk limbs). The limb on the somite adjacent to the posterior somite on this remipede is expected to be reorganized, with a protopod of coxapod and basipod, but fewer than 3 exopodal and fewer than 4 endopodal segments (e.g., Fig. 4, specimen with 23 trunk limbs; Figs. 5F–5G, specimen with 34 trunk limbs).

Copepods have trunk limbs very similar to remipedes, and provide an interesting comparison to remipedes. Copepod trunk somites are divided into an anterior set of seven (TS 1–7) that bear limbs and a posterior set of four limbless somites (TS 8–11). Like remipedes, the anterior somite of copepods bears a maxilliped whose structure differs significantly from the other trunk limbs. The limbs on TS 2–6 often are quite similar to one another, flattened anterio-posteriorly, like those of remipedes. Based on the presence of arthrodial membranes, these limbs are made up of two protopodal segments, coxapod and basipod, and rami of three exopodal segments and three endopodal segments. A contralateral pair of limbs is united by a transverse sclerotized bar fused to the coxapod of each limb and articulating with the sternum, a morphology similar to the sternal bar of remipedes. TL 2–7 of copepods begin development as a setose, usually bilobate bud; these buds usually originate ventrally. However, unlike remipedes, presence of the transverse sclerotized bar on this bud is not clear. During the following molt, a bud pair is reorganized into a transformed, segmented limb, and subsequent molts usually add segments and setae in an orderly way (Ferrari & Dahms, 2007). TL 7 of copepods remains a bud and is never reorganized. It is associated with the genital opening of both sexes on the terminal adult stage.

Both rami on trunk limbs posterior to the maxilliped of remipedes are patterned proximally from the distal segment of the ramis. The rami of TL 2–7 of copepods also appear to be patterned proximally from the distal segment, although the distal ramal segment on copepods may be a segment complex, so new segments actually may be added either proximally or distally from a point in the middle of the ramus (Ferrari & Dahms, 2007).

On remipedes, setae are added to a new segment on a trunk limb several molts after the arthrodial membrane delineates the newly formed segment. On TL 2–6 of copepods a single seta, the formation seta, usually is added proximally on the distal segment before a new arthrodial membrane delineates the new segment (Ferrari & Ambler, 1992). The sternal or transverse ligamentous bar of remipedes is similar in location and function to the transverse sclerotized bar of copepods. Both transverse structures articulate with the sternum and both unite only the coxapod of contralateral limb pairs. The sternal bar of remipedes and the transverse sclerotized bar of copepods have not been considered homologous, but they have not been studied comparatively in detail. However, their ventral location between contralateral coxapods, the similarity in the way limbs of both groups develop, and similarities of naupliar structures (Ferrari et al., 2011) suggests that homology seems reasonable.

On remipedes the number of setae present on the rami of limbs along the anterior/posterior axis shows a similar pattern. Setal numbers initially increase, sometimes rather sharply, reaching the maximum between TL 8–12; the number of setae then decreases, sometimes rather sharply, to the limb on the posterior segment. This is true for the total number of setae on a ramus (Figs. 7C–7F) as well as the number on individual ramal segments, regardless of the number of somites comprising the body, i.e., the stage of development. The only exception to this distribution is found for the proximal segment of the rami that bears only a few setae (Tables 2 and 3).

Setal numbers also have been studied on the juveniles and adult of *Dioithona oculata* a podoplean copepod on which TL 2–5 are well-developed and 3-segmented (Ferrari & Ambler, 1992). The number of setae on these rami is much smaller than for remipedes, and these setae are usually arranged along the dorsal and ventral faces of the rami rather than on distinctive groups. However, in general the number of setae on TL 2–5 increases from TL 2 to TL 3, and then decreases on TL 4–TL 5. This pattern is found on four developmental stages, viz. juvenile copepodid III through copepodid V, and adult copepodid VI (Ferrari & Ambler, 1992: Table 4). So despite having fewer limbs with fewer setae, changes in number of setae on the limbs of copepods is similar to that found on remipede limbs, reaching a maximum between the anterior and posterior limbs.

Among other crustaceans, many branchiopods have anterio-posterior flattened limbs with a large number of setae, and notostracan branchiopods also have a larger number of trunk limbs than remipedes (Schram, 1986; Brusca, Moore & Shuster, 2016). Among malacostracans, phyllocarida have eight anterior trunk limbs (TL 1–8) of similar morphology, and many eumalacostracans have five posterior trunk limbs (TL 9–13) that are flattened anterio-posteriorly and bear many setae (Schram, 1986; Brusca, Moore & Shuster, 2016). These crustaceans have yet to be analyzed for setal numbers on these anterio-posterior set of limbs, and it will be interesting to discover whether the peculiar change in setal numbers found here can also are observed in these crustacean groups.

The vector analysis here demonstrates that the setation is determined by the limb and group identity, not requiring additional interaction terms. At that, the comparison of the limb vector profile demonstrates that setation patterns and, possibly, development of several first somites and the remaining somites are different, as the former are standard, whereas the latter seemingly depend on their age. The simplest explanation for these observations is that buds of setae are set in a standard form, and then develop in a certain order with each new molt. At that, application of the vector formula to distinguish a remipede stages using limited information of this study, as well any prediction of the maximum number of the developmental stages, requires additional investigation. This vector analysis is the first example of such an analysis formalizing a general pattern of developmental changes in serially homologous structures of an arthropod. The results show that the development of the setation of the trunk limbs correlates well with the number of somites and the position of the limb along the anterior-posterior axis.

In general, comparative analysis of many described species of remipedes is limited by lack of detailed information about segmentation as well as setation of the limbs. This information is essential for identification of developmental stage, as well as the species identity. The standard minimum description of a remipede should include homonomous trunk limbs on the following somites: the most anterior, the TL8 with gonopore, the most posterior limb with completely segmented rami (2 - 3 - 4) and any incompletely segmented posterior limbs, including limb buds.

##  Supplemental Information

10.7717/peerj.2305/supp-1 Table S1 Segmentation of trunk limbs, posterior to the maxilliped, of examined specimens of *Speleonectes tulumensis* Yager, 1987 Abbreviation: TL –thoracopod, 3–4 = 3 segmented exopod and 4 segmented endopod; bud, bilobe limb bud; a, anal somite bearing caudal rami.

10.7717/peerj.2305/supp-2 Table S2 *Speleonectes tulumensis* Yager, 1987 Specimen B. Number of setal elements in each of 20 groups of the thoracopods 35–37. Abbreviations: (-), the segment is not distinguishing; r, right leg; l, left leg.

10.7717/peerj.2305/supp-3 Table S3 *Speleonectes tulumensis* Yager, 1987 Specimen C. Number of setal elements in each of 20 groups of the thoracopods 34–36. Abbreviations: (-), the segment is not distinguishing; r, right leg; l, left leg.

10.7717/peerj.2305/supp-4 Table S4 *Speleonectes tulumensis* Yager, 1987 Specimen D. Number of setal elements in each of 20 groups of the thoracopods 34–35. Abbreviations: (-), the segment is not distinguishing; r, right leg; l, left leg.

10.7717/peerj.2305/supp-5 Table S5 *Speleonectes tulumensis* Yager, 1987 Specimen E. Number of setal elements in each of 20 groups of the thoracopods 31–33. Abbreviations: (-), the segment is not distinguishing; r, right leg; l, left leg.

10.7717/peerj.2305/supp-6 Supplemental Information 1 Supplemental Script Script for decomposition of the setation matrix into the trunk limb and group setation vectors.

## Additional Information and Declarations

### Data Availability

The following information was supplied regarding data availability: The raw data has been supplied as Supplemental File.
