# Supplementary material for: Changes in segmentation and setation along the anterior/posterior axis of the homonomous trunk limbs of a remipede (Crustacea, Arthropoda)
Source: PeerJ. 2016 Aug 10;4:e2305. doi: 10.7717/peerj.2305 (PMC4991865; doi:10.7717/peerj.2305)
Supplement: Table S1 — Abbreviation: TL –thoracopod, 3–4 = 3 segmented exopod and 4 segmented endopod; bud, bilobe limb bud; a, anal somite bearing caudal rami. [file peerj-04-2305-s001.docx]

|  | TLs 2-20 | TL 21 | TL 22 | TL 23 | TL 24 | TLs 25-31 | TL 32 | TL 33 | TL 34 | TL 35 | TL 36 | TL 37 | TL 38 | TL 39 |  |
| --- | --- | --- | --- | --- | --- | --- | --- | --- | --- | --- | --- | --- | --- | --- | --- |
| A | 3-4 | 3-4 | 3-4 | 3-4 | 3-4 | 3-4 | 3-4 | 3-4 | 3-4 | 3-4 | 3-4 | 3-4 | 3-4 | bud | a |
| B | 3-4 | 3-4 | 3-4 | 3-4 | 3-4 | 3-4 | 3-4 | 3-4 | 3-4 | 3-4 | 3-4 | 1-2 | a |  |  |
| C | 3-4 | 3-4 | 3-4 | 3-4 | 3-4 | 3-4 | 3-4 | 3-4 | 3-4 | 3-4 | bud | a |  |  |  |
| D | 3-4 | 3-4 | 3-4 | 3-4 | 3-4 | 3-4 | 3-4 | 3-4 | 3-4 | 3-4 | a |  |  |  |  |
| E | 3-4 | 3-4 | 3-4 | 3-4 | 3-4 | 3-4 | 3-3 | 1-2 | a |  |  |  |  |  |  |
| F | 3-4 | 3-3 | 1-2 | bud | a |  |  |  |  |  |  |  |  |  |  |
