# Supplementary material for: Changes in segmentation and setation along the anterior/posterior axis of the homonomous trunk limbs of a remipede (Crustacea, Arthropoda)
Source: PeerJ. 2016 Aug 10;4:e2305. doi: 10.7717/peerj.2305 (PMC4991865; doi:10.7717/peerj.2305)
Supplement: Table S5 — Specimen E. Number of setal elements in each of 20 groups of the thoracopods 31–33. Abbreviations: (-), the segment is not distinguishing; r, right leg; l, left leg. [file peerj-04-2305-s005.docx]

|  | 1 | 2 | 3 | 4 | 5 | 6 | 7 | 8 | 9 | 10 | 11 | 12 | 13 | 14 | 15 | 16 | 17 | 18 | 19 | 20 |
| --- | --- | --- | --- | --- | --- | --- | --- | --- | --- | --- | --- | --- | --- | --- | --- | --- | --- | --- | --- | --- |
| 31r | 0 | 0 | 1 | 0 | 0 | 1 | 2 | 3 | 0 | 0 | 0 | 1 | 0 | 0 | 0 | 0 | 0 | 0 | 1 | 2 |
| 31l | 0 | 0 | 1 | 0 | 0 | 1 | 4 | 2 | 0 | 0 | 0 | 1 | 0 | 0 | 0 | 0 | 0 | 0 | 2 | 2 |
| 32r | 0 | 0 | (-) | (-) | (-) | (-) | 2 | 2 | 0 | 0 | 0 | 1 | 0 | 0 | (-) | (-) | (-) | (-) | 1 | 2 |
| 32l | 0 | 0 | (-) | (-) | (-) | (-) | 2 | 2 | 0 | 0 | 0 | 1 | 0 | 0 | (-) | (-) | (-) | (-) | 1 | 2 |
| 33l | (-) | (-) | (-) | (-) | (-) | (-) | 1 | 1 | (-) | (-) | (-) | (-) | (-) | (-) | (-) | (-) | (-) | (-) | 1 | 1 |
