# Supplementary material for: Changes in segmentation and setation along the anterior/posterior axis of the homonomous trunk limbs of a remipede (Crustacea, Arthropoda)
Source: PeerJ. 2016 Aug 10;4:e2305. doi: 10.7717/peerj.2305 (PMC4991865; doi:10.7717/peerj.2305)
Supplement: Supplemental Information 1 — Script for decomposition of the setation matrix into the trunk limb and group setation vectors. [file peerj-04-2305-s006.docx]

## Supplemental script

Script for decomposition of the setation matrix into the trunk limb and group setation vectors.

import numpy

import math

def get_norm (data, x, y):

norm = 0

for i in range(n):

for j in range(m):

norm += (x[i]*y[j] - float(data[i][j]))**2

norm = math.sqrt(norm)

return norm

def decomposition (data):

dataTdata = [[0 for j in range (m)] for i in range (m)]

for i in range(m):

for j in range(m):

for k in range(n):

dataTdata[i][j] += (data[k][i])*(data[k][j])

eigenvectors_y = numpy.linalg.eig(dataTdata)[1]

good_norm = 100000000

for i in range(0, m):

vector_y = [0 for k in range(m)]

for j in range(m):

vector_y[j] = eigenvectors_y[j][i]

vector_x = [0 for i in range(n)]

for i in range(n):

for j in range(m):

vector_x[i] += (data[i][j])*(vector_y[j])

new_norm = get_norm (data, vector_x, vector_y)

if new_norm < good_norm:

x = vector_x

y = vector_y

good_norm = new_norm

return x, y, good_norm
